# A novel continuous-progression CKM model combined with multi-omics data integration identifies lipid metabolic drivers of disease progression

**DOI:** 10.3389/fendo.2026.1910505

**Published:** 2026-07-29

**Authors:** Hai-Lun Ye, Ya-Ni Wang, Gang-Ao Li, Xing-Hui Jin, Yang Li, Ying-Hua Jin

**Affiliations:** 1 Key Laboratory for Molecular Enzymology and Engineering of the Ministry of Education, School of Life Sciences, Jilin University, Changchun, China; 2 Laboratory of Ginseng and Human Health, Jilin University, Changchun, China; 3 Ginseng Research Institute, Jilin University, Changchun, China

**Keywords:** animal model, cardiovascular–kidney–metabolic (CKM), single-cell transcriptomic, spatial transcriptomics, virtual drug screening

## Abstract

**Introduction:**

Cardiovascular–kidney–metabolic (CKM) syndrome represents an emerging systemic disorder characterized by intertwined metabolic dysfunction, chronic kidney disease, and cardiovascular injury, yet robust preclinical models and mechanistic insights remain limited.

**Methods:**

Here, we established a progressive rat model of CKM syndrome by combining high-fat/high-sucrose exposure with adenine-induced renal stress, capturing temporal changes from early metabolic dysregulation to advanced multi-organ injury. Integrative multi-omics analyses incorporating network toxicology, single-cell RNA sequencing, and spatial transcriptomics were performed to investigate molecular features associated with CKM progression. Finally, AI-assisted virtual screening coupled with molecular docking was conducted to identify potential multi-target therapeutic candidates.

**Results:**

Longitudinal phenotyping revealed a temporal pattern in which early metabolic abnormalities preceded more prominent renal and cardiovascular impairment. Integrative multi-omics analyses incorporating network toxicology, single-cell RNA sequencing, and spatial transcriptomics consistently converged on lipid metabolic dysregulation as a prominent molecular feature associated with CKM progression. PPARγ, ESR1, and FASN were identified as candidate regulatory nodes associated with CKM-related lipid remodeling. Single-cell analyses revealed enrichment of lipid metabolic programs in renal epithelial compartments, particularly proximal tubular cells, suggesting their potential involvement in CKM-associated renal metabolic remodeling. Spatial transcriptomics further revealed patterns consistent with ectopic adipocyte infiltration, lipid-associated niche remodeling, increased PPARγ/FASN expression, and reduced ESR1 expression in diseased kidneys. AI-assisted virtual screening coupled with molecular docking identified BRD-K26818574 as a potential multi-target therapeutic candidate.

**Discussion:**

Collectively, this study establishes a translational CKM model and highlights renal lipotoxic remodeling as a potential therapeutic target in CKM progression.

## 1 Introduction

Cardiovascular disease (CVD) (1, 2), chronic kidney disease (CKD) (3, 4), obesity (5), insulin resistance, and type 2 diabetes (6, 7) mellitus are increasingly interconnected global health challenges that collectively account for substantial morbidity, mortality, and healthcare expenditure worldwide. Traditionally, these disorders have been investigated as independent clinical entities. However, accumulating evidence indicates that they are biologically intertwined through shared metabolic, inflammatory, neurohormonal, and hemodynamic mechanisms (8–13). Recognizing this systemic interdependence, the American Heart Association recently introduced the concept of cardiovascular–kidney–metabolic (CKM) syndrome (14), redefining these conditions as components of a continuous multisystem disorder rather than isolated diseases.

CKM syndrome describes a progressive pathophysiological continuum in which excess adiposity, insulin resistance, dyslipidemia, hypertension, renal dysfunction, and cardiovascular injury mutually reinforce one another (14, 15). Persistent metabolic stress promotes endothelial dysfunction, chronic low-grade inflammation (16, 17), oxidative stress (18, 19), and activation of the renin–angiotensin–aldosterone system (RAAS) (20, 21), thereby accelerating structural and functional deterioration in both the heart and kidneys. Conversely, renal dysfunction (22, 23) amplifies systemic metabolic imbalance and cardiovascular burden through sodium retention, toxin accumulation, neurohormonal activation, and altered substrate metabolism. This maladaptive bidirectional crosstalk establishes a self-sustaining pathological cardiorenal-metabolic cycle.

Despite growing clinical recognition, mechanistic understanding of CKM syndrome remains limited. Most current evidence derives from epidemiological studies, risk stratification models, and narrative reviews, whereas experimental studies that capture the integrated and progressive nature of CKM are scarce. Existing animal models typically focus on isolated obesity, diabetes, heart failure, or CKD phenotypes, and therefore fail to recapitulate the full CKM continuum. This translational gap has hindered the identification of shared upstream drivers and the development of targeted therapies.

In the present study, we established a progressive rat model of CKM syndrome by combining high-fat/high-sucrose exposure with adenine-induced renal stress, thereby reproducing the transition from early metabolic dysfunction to overt multi-organ injury. We further integrated systems biology, network toxicology, single-cell RNA sequencing, spatial transcriptomics, and AI-assisted virtual drug screening to define the molecular architecture of CKM progression. Our findings identify renal lipid metabolic reprogramming as a central pathological feature of CKM and nominate actionable regulatory targets with potential translational relevance.

## 2 Materials and methods

### 2.1 Animals

**Figure 1:**
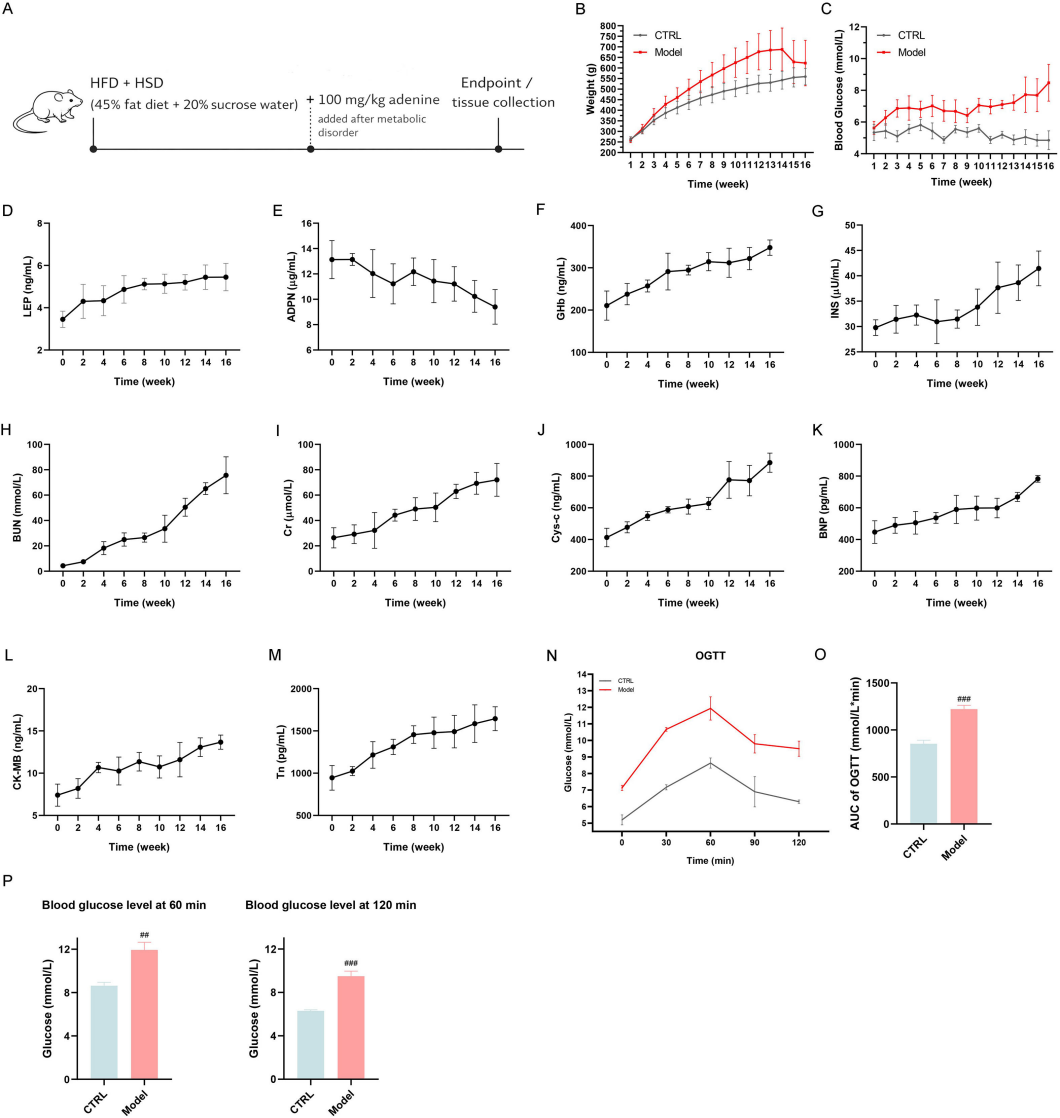
Establishment of the dual-stage cardiorenal metabolic (CKM) model. **(A)** Schematic diagram of the experimental design and model construction protocol (each group n=7). **(B)** Changes in body weight of rats during model induction. **(C)** Temporal changes in blood glucose levels throughout the model construction phase. **(D–M)** Serum concentrations of leptin (LEP), adiponectin (ADPN), glycated hemoglobin (GHb), insulin (INS), blood urea nitrogen (BUN), creatinine (Cr), cystatin C (Cys-c), brain natriuretic peptide (BNP), creatine kinase-MB (CK-MB), and troponin (Tn) measured during model development. **(N)** Oral glucose tolerance test (OGTT) performed at week 10. **(O)** Statistical summary of the area under the curve (AUC) derived from the OGTT. **(P)** Quantification of blood glucose levels at the 60-minute peak and at the 120-minute endpoint during the OGTT. ^##^p < 0.01, ^###^p < 0.001vs.CTRL.

Male Wistar rats (6–8 weeks old, specific pathogen-free [SPF] grade) were purchased from Beijing Vital River Laboratory Animal Technology Co., Ltd. (Beijing, China). The animals were housed in the Animal Experiment Center of the College of Life Sciences, Jilin University (Changchun, China). Housing conditions were maintained at an SPF level with *ad libitum* access to food and water, an ambient temperature of 22 ± 2 °C, and relative humidity of 50 ± 5%. Rats were randomly assigned to groups, and the experimental procedures were conducted according to the schedule illustrated in **Figure 1A**. After acclimatization, rats were randomly assigned to experimental groups according to the study design. To establish the CKM model, animals received a high-fat diet (45%) together with 20% sucrose-supplemented drinking water for induction of systemic metabolic dysfunction. After confirmation of metabolic abnormalities, adenine was administered to accelerate renal injury and promote progression toward advanced cardiorenal dysfunction. Age-matched control rats received standard chow and normal drinking water. After the adaptation period, 7 rats were randomly selected to serve as the healthy control group, while the remaining animals underwent modeling procedures to induce the disease state. Upon successful establishment of the metabolic disorder, another 7 rats were randomly chosen for interim sampling, and the rest continued with further modeling, and the experiment was terminated in a timely manner upon observation of a marked deterioration in the animals’ overall living conditions. This study employed a double-blind design: in addition to the experimental animals being unaware of the intervention, the group allocation was also concealed from the behavioral observers and data analysts.

### 2.2 ELISA analysis of serum indicators

Blood samples were collected using capillary tubes. Serum was separated by centrifugation at 12,000 × *g*. Serum levels of leptin (LEP), adiponectin (ADPN), glycated hemoglobin (GHb), insulin (INS), blood urea nitrogen (BUN), creatinine (Cr), cystatin C (Cys-C), brain natriuretic peptide (BNP), creatine kinase-MB (CK-MB), and troponin (Tn) were measured by Changchun Shixu Biotechnology Co., Ltd. (Changchun, China) using commercial ELISA kits in strict accordance with the manufacturer’s instructions.

### 2.3 Oral glucose tolerance test

Following a fasting period of 10–12 h, baseline fasting blood glucose levels were measured. Rats were subsequently administered a glucose solution (2.0 g/kg body weight) via oral gavage. Blood glucose levels were measured at 30, 60, 90, and 120 min post-gavage. The area under the blood glucose curve (AUC) was calculated using the trapezoidal rule.

The area under the curve (AUC) for the glycemic response was calculated using the trapezoidal rule. The following formula was applied:

AUC (mmol/L·min) = 15 × (G_0_ + 2G_30_ + 2G_60_ + 2G_90_ + G_120_)

where G_0_, G_30_, G_60_, G_90_, and G_120_ represent the blood glucose concentrations (mmol/L) at 0, 30, 60, 90, and 120 minutes, respectively. This formula is derived from the weighted sum of the mean glucose values between consecutive time points, all of which are equally spaced at 30-minute intervals.

### 2.4 Echocardiographic assessment

Cardiac structure and systolic function were evaluated using transthoracic echocardiography at study endpoint. Left ventricular ejection fraction (EF) and fractional shortening (FS) were recorded and analyzed by investigators blinded to group allocation.

### 2.5 Histopathological analysis

Fresh tissue samples were fixed in 4% phosphate-buffered paraformaldehyde for 24 h. Following fixation, the tissues were dehydrated through a graded sucrose gradient (15%, 20%, and 25%) and incubated at 4 °C for 24 h. The fixed tissue blocks were embedded in paraffin using standard protocols. Sections (4-μm thick) were cut and stained with hematoxylin and eosin (H&E). Histopathological changes were observed and imaged using an Olympus microscope (Olympus, Tokyo, Japan).

### 2.6 scRNA-seq data acquisition and processing

The single-cell dataset was derived from GSE GSE142153 in the GEO database (10 normal samples, 23 disease samples) and analyzed using R 4.1.3 and the “Seurat” package: High-quality cells were first filtered via cytoplasmic quality control (mitochondrial gene ratio<20%, red blood cell gene ratio<3%, cell UMI count 200∼25000, gene count 200∼5000). Data normalization was performed using the NormalizeData function, 2000 highly variable genes were selected with the FindVariableFeatures function, and the ScaleData function (parameter: vars.to.regress = c(S.Score, G2M.Score)) was used to eliminate cell cycle effects. Batch effects were corrected using the “Harmony” package, dimensionality reduction was performed with the TSNE function, and clustering was conducted using the Louvian algorithm in Seurat. Finally, the FindAllMarkers function was used to calculate inter-cluster differentially expressed genes (DEGs) with the following criteria: p<0.05, log_2_FC>0.25, and expression ratio>0.1. Spatial transcriptomic samples from GSE183456 were obtained from the GEO database for subsequent analyses.

### 2.7 Acquisition of lipid metabolism-related genes

Gene sets associated with lipid metabolism were retrieved from the Molecular Signatures Database (MSigDB), specifically “GOBP_LIPID_METABOLIC_PROCESS” (GO:0006629) and “GOBP_FATTY_ACID_METABOLIC_PROCESS” (GO:0006631). The corresponding gene set files were downloaded directly from the database, and the gene sets were integrated for subsequent lipid metabolism scoring analysis.

### 2.8 Cell annotation analysis

Clustering results were annotated based on classical cell type marker genes, including specific markers for 10 cell types (e.g., epithelial cells: endothelial cells; adipocytes: T cells). After annotation, TSNE plots were generated using the DimPlot function,

### 2.9 Single-cell pseudotime analysis

Single-cell pseudotime trajectory inference was performed using the “Slingshot” package in R. Following the extraction of the target cell subset data, normalized expression matrices and dimensionality reduction coordinates (PCA or UMAP results by default) were obtained from “Seurat” or “SingleCellExperiment” objects. The slingshot function was subsequently applied to construct a minimum spanning tree (MST) based on the specified reduced-dimensional space and to fit smooth curves, thereby inferring cell differentiation trajectories. When prior biological knowledge regarding the origin of differentiation was available, the starting cluster was specified using the start.clus parameter; otherwise, the starting point was determined by diffusion component analysis or established biological priors. Pseudotime values for individual cells along each lineage were extracted using the slingPseudotime function, and fitted curve coordinates were obtained via the slingCurves function for downstream trajectory visualization and analysis of dynamic gene expression patterns.

### 2.10 Cell–cell communication analysis

Cell–cell communication analysis was conducted using the “CellChat” R package. Normalized gene expression matrices were imported into the CellChat object. Data preprocessing was performed using the identify Over Expressed Genes, identify Over Expressed Interactions, and project Data functions. Ligand–receptor interactions were identified and quantified using the compute Commun Prob, filter Communication, and compute CommunProb Pathway functions. Finally, the aggregateNet function was employed to construct and visualize the cell–cell communication network.

### 2.11 Artificial intelligence-driven drug prediction

The DrugRefLector framework (24), which leverages active learning with transcriptomic data, was utilized to identify regulatory factors associated with disease phenotypes. Expression microarray datasets GSE30528, GSE30529, and GSE142153, retrieved from the Gene Expression Omnibus (GEO) database, were input into the DrugRefLector deep learning model to screen for candidate therapeutic compounds based on compound-induced phenotypic activity.

### 2.12 Molecular docking

The chemical structures of compounds selected from virtual screening were obtained from the PubChem database. Molecular docking simulations were performed using AutoDock (v4.2.6) to evaluate the binding affinities and interaction modes of these compounds with the active sites of PPARγ (PDB ID: 8WFE), ESR1 (PDB ID: 3OS9), and FASN (AF-P49327). Following standard protocols, non-essential residues were removed from the protein structures, and the active site coordinates of the investigated proteins were defined. The docking results were visualized using PyMOL software.

### 2.13 Acquisition of toxicology targets

The SMILES strings of palmitic acid, sucrose, and adenine were retrieved from the PubChem database (https://pubchem.ncbi.nlm.nih.gov/). Potential targets for these compounds were predicted using TargetNet (http://targetnet.scbdd.com/), SwissTargetPrediction (http://swisstargetprediction.ch/), the Similarity Ensemble Approach (SEA; https://sea.bkslab.org/), and ChEMBL (https://www.ebi.ac.uk/chembl/).

Disease targets associated with cardiometabolic kidney (CKM) syndrome were collected from the following databases: GeneCards (https://www.genecards.org/), the Therapeutic Target Database (TTD; http://db.idrblab.net/ttd/), DisGeNET (https://www.disgenet.org/search), the Comparative Toxicogenomics Database (CTD; http://ctdbase.org/search/), and the Online Mendelian Inheritance in Man (OMIM; https://omim.org/).

### 2.14 Statistical analysis

Experimental data are presented as the mean ± standard deviation (SD). Statistical analyses were performed using GraphPad Prism 8 software. Comparisons between two groups were conducted using Student’s t-test. A *P*-value< 0.05 was considered to indicate a statistically significant difference.

## 3 Results

### 3.1 A progressive rat model recapitulates staged CKM evolution from metabolic dysfunction to overt cardiorenal injury

Given the multifactorial complexity and multi-organ crosstalk inherent to cardiovascular–kidney–metabolic (CKM) syndrome, the development of experimental models that recapitulate its progressive pathophysiology remains essential for mechanistic investigation. In this study, we established a composite rat model by combining a high-fat/high-sucrose dietary intervention with adenine administration, aiming to mimic the transition from early metabolic dysregulation to advanced multi-organ injury. Specifically, rats were subjected to a 45% high-fat diet and 20% sucrose-supplemented drinking water to induce early metabolic disturbances. Upon confirmation of metabolic impairment, adenine was introduced to exacerbate renal burden and accelerate the progression toward systemic organ damage (**Figure 1A**).

Longitudinal monitoring revealed progressive increases in body weight and fasting blood glucose levels throughout the dietary induction period, indicating successful establishment of systemic metabolic dysfunction (**Figures 1B, C**). Circulating metabolic biomarkers, including leptin, adiponectin, glycated hemoglobin, and insulin, exhibited pronounced temporal dysregulation (**Figures 1D–G**). Oral glucose tolerance testing further demonstrated impaired glucose homeostasis and insulin sensitivity (**Figures 1N–P**).

Notably, several biomarkers reached a relative plateau during weeks 8–10, suggesting stabilization of the early metabolic disturbance. Based on this temporal profile, adenine administration was initiated at week 10 to promote progression toward advanced CKM pathology.

**Figure 2:**
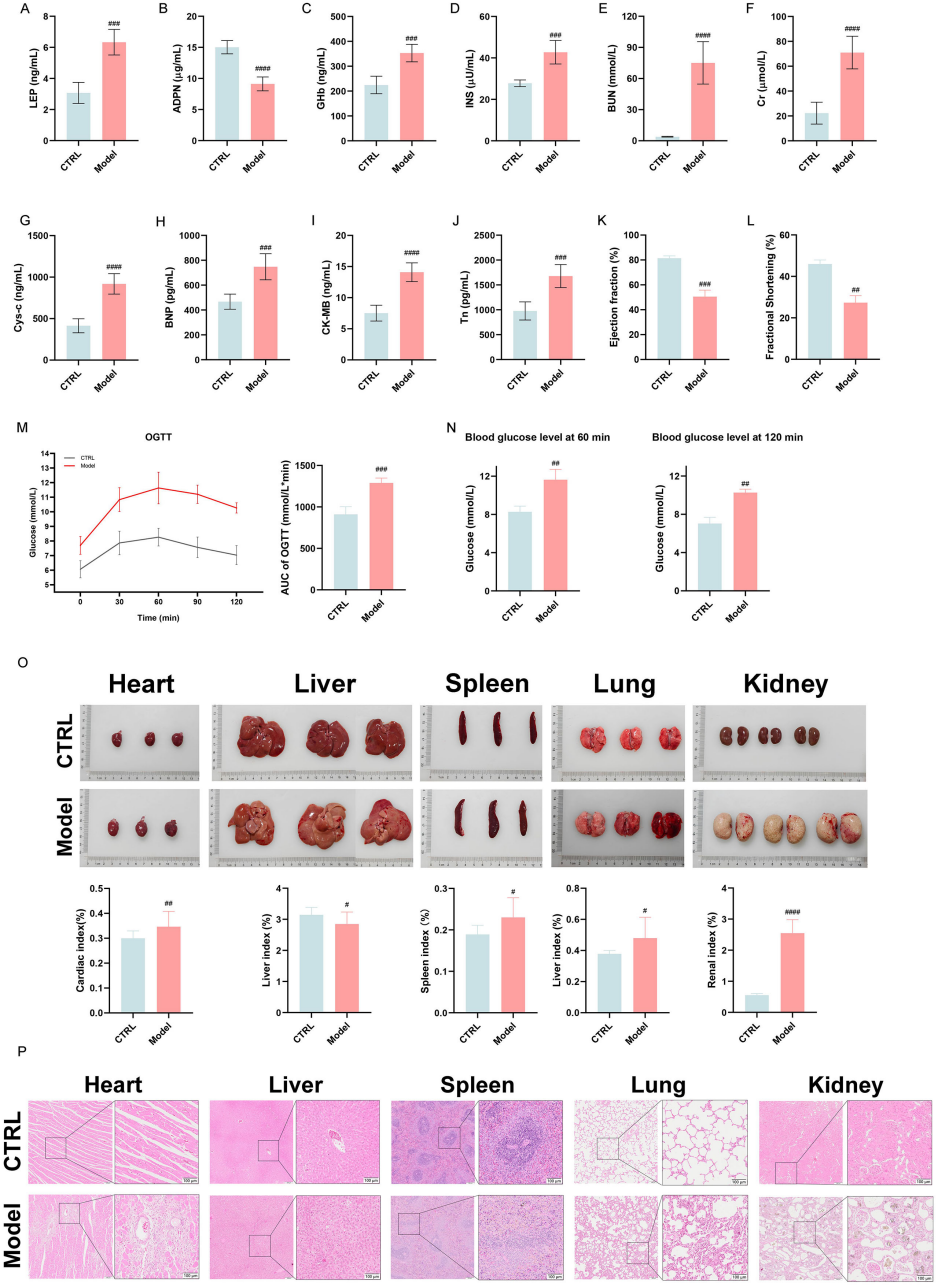
Multi-organ injury induced by CKM model establishment. **(A–J)** Serum levels of LEP, ADPN, GHb, INS, BUN, Cr, Cys-c, BNP, CK-MB, and Tn in each experimental group at the study endpoint. **(K)** Statistical analysis of ejection fraction (EF) across groups at endpoint. **(L)** Statistical analysis of fractional shortening (FS) across groups at endpoint. **(M)** Endpoint oral glucose tolerance test results and corresponding AUC analysis. **(N)** Quantification of blood glucose peak at 60 min and endpoint glucose levels at 120 min during the final OGTT. **(O)** Histological assessment of morphological differences in the heart, liver, spleen, lung, and kidney, accompanied by statistical analysis of organ indices. **(P)** H&E of heart, liver, spleen, lung, and kidney. ^#^p < 0.05, ^##^p < 0.01, ^###^p < 0.001, ^####^p < 0.0001 vs. CTRL.

Following adenine administration, renal injury markers (BUN, creatinine, cystatin C) and cardiac injury markers (BNP, CK-MB, troponin) deteriorated rapidly (**Figures 1H–M**). A significant decline in body weight was observed during the late phase, consistent with systemic decompensation. Endpoint echocardiography revealed reduced ejection fraction (EF) and fractional shortening (FS), confirming impaired cardiac systolic function (**Figures 2K, L**).

Histopathological examination demonstrated structural injury across multiple organs (**Figures 2O, P**), with particularly severe injury observed in renal and cardiac tissues, supporting successful establishment of multisystem damage (**Figures 2A–N**).

### 3.2 Convergent systems analyses nominate lipid dysregulation as a central CKM driver

To identify molecular mechanisms underlying CKM progression, representative exposure factors used in model construction (palmitic acid, sucrose, and adenine) were subjected to integrative network toxicology analysis.

**Figure 3:**
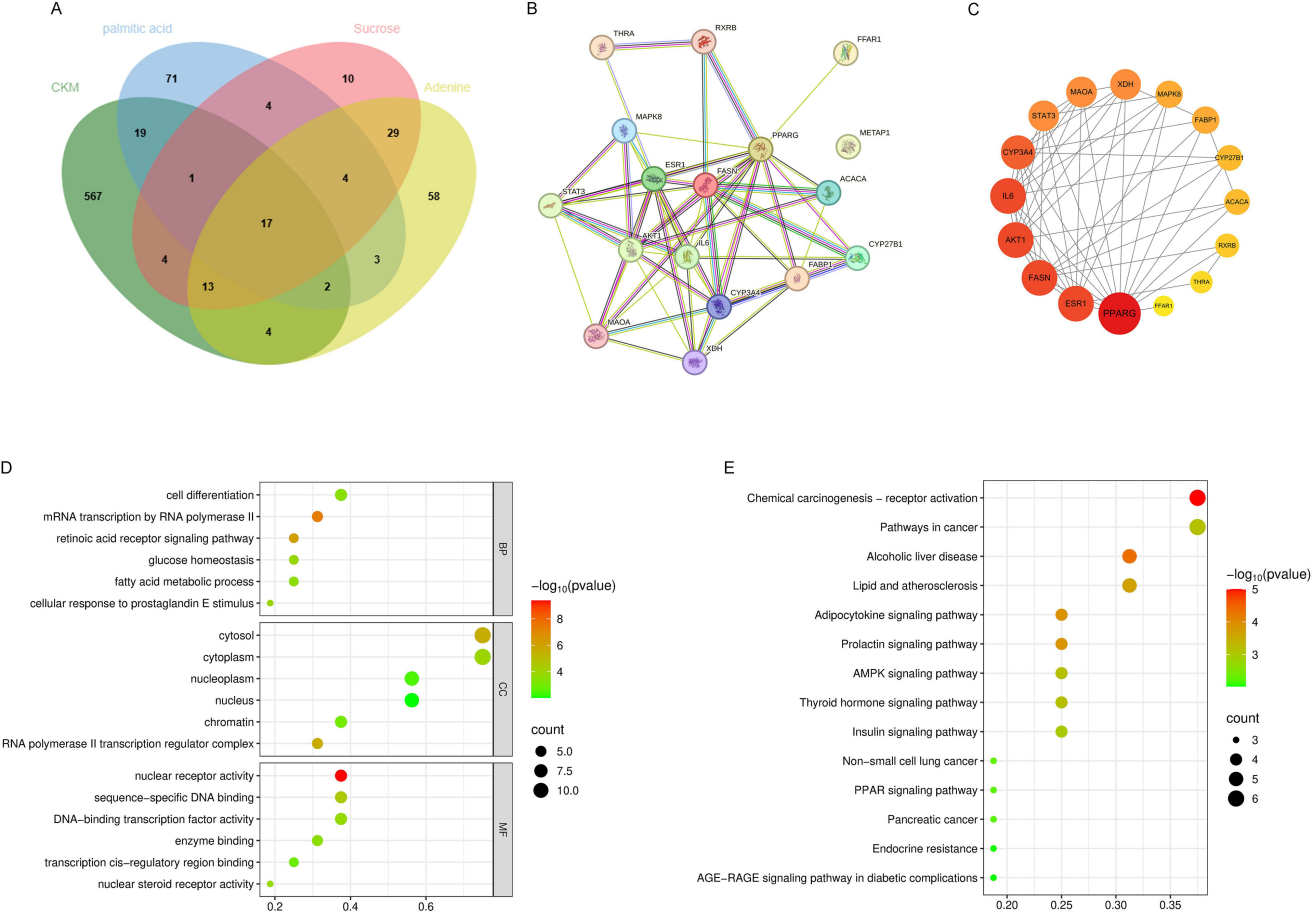
Network toxicology analysis of interventions based on model construction. **(A)** Venn diagram illustrating the direct targets overlapping between adenine, sucrose, palmitic acid, and CKM. **(B)** Protein-protein interaction (PPI) network constructed from the core intersecting targets. **(C)** Ranking of core intersecting targets based on node degree within the PPI network. **(D)** Gene Ontology (GO) enrichment analysis of the core target set. **(E)** Kyoto Encyclopedia of Genes and Genomes (KEGG) pathway enrichment analysis of the core target set.

Seventeen intersecting genes were identified between compound-associated targets and CKM-related disease genes (**Figure 3A**). Protein–protein interaction network analysis followed by topological ranking identified Peroxisome Proliferator-Activated Receptor Gamma (PPARγ), Estrogen Receptor 1 (ESR1), and Fatty Acid Synthase (FASN) as the three highest-priority hub genes (**Figures 3B, C**), suggesting central regulatory roles.

Gene Ontology enrichment demonstrated significant involvement in glucose homeostasis, fatty acid metabolism, lipid storage, and hormone-responsive pathways (**Figure 3D**). KEGG pathway analysis further revealed enrichment in the PPAR signaling pathway, lipid and atherosclerosis, and adipocytokine signaling pathways (**Figure 3E**). These convergent analyses consistently position dysregulated lipid metabolism as a shared upstream determinant of CKM progression.

### 3.3 Single-cell transcriptomics highlights the critical role of lipid metabolism in disease progression

**Figure 4:**
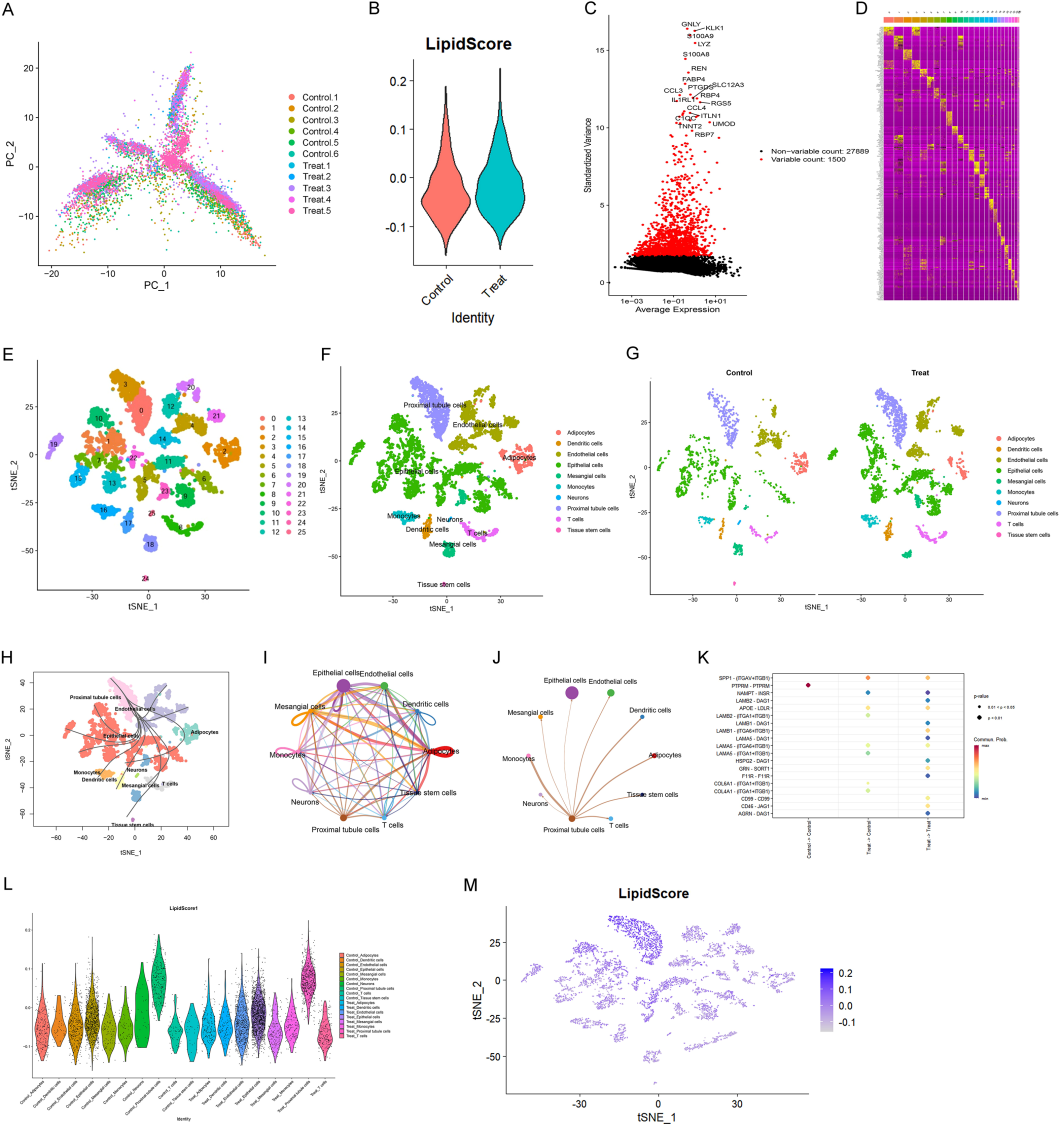
Single-cell transcriptomic profiling of renal tissue in a CKM model based on GEO datasets. **(A)** Principal component analysis (PCA) plot illustrating sample clustering and distribution. **(B)** Violin plots depicting the expression landscape of lipid metabolism-associated genes across different experimental groups. **(C)** Analysis of differentially expressed genes (DEGs) in the disease group. **(D)** Unsupervised clustering classification of single-cell transcriptomic data. **(E)** t-distributed stochastic neighbor embedding (t-SNE) visualization of identified cell clusters. **(F)** Cell-type annotation and functional identification of distinct populations. **(G)** Distribution and proportional representation of various cell types among different groups. **(H)** Pseudotime trajectory analysis revealing cell lineage differentiation dynamics. **(I)** Global intercellular communication network analysis. **(J)** Specific analysis of intercellular communication pathways in proximal tubule epithelial cells. **(K)** Identification of highly significant ligand-receptor interaction pairs. **(L)** Expression patterns of lipid metabolism-related genes across distinct annotated cell clusters. **(M)** Spatial distribution and expression landscape of lipid metabolism-associated genes within the disease group.

Given that the kidney exhibited the most pronounced pathological changes during model establishment, subsequent analyses were focused on renal tissue to further investigate the critical role of lipid metabolism in kidney injury during disease progression. Analysis of single-cell transcriptomic data from the GEO database revealed a substantial remodeling of the global transcriptional landscape in diseased kidneys (**Figure 4A**). Gene set scoring for lipid metabolism-related pathways demonstrated a significant increase in lipid metabolic activity in the disease group (**Figure 4B**). Differential expression analysis further showed that among the top 15 most significantly altered genes, several key regulators of lipid metabolism—such as Retinol Binding Protein 4 (RBP4), Fatty Acid Binding Protein 4 (FABP4), and Retinol Binding Protein 7 (RBP7)—were prominently enriched (**Figure 4C**).

To characterize cellular heterogeneity, clustering and cell-type annotation were performed (**Figures 4D–F**). The results indicated a marked expansion of epithelial cells and proximal tubular cell populations (**Figure 4G**), suggesting severe disruption of renal homeostasis accompanied by an increase in lipid metabolism-active cell types. Further pseudotime trajectory and cell–cell communication analyses revealed that proximal tubular cells are positioned at the early stage of the disease progression trajectory and exhibit distinct intercellular communication patterns (**Figures 4H–K**), highlighting their potential driving role in disease development. Moreover, lipid metabolism scoring across different cell types showed a consistent increase in all cell populations in the disease group, with the most pronounced elevation observed in proximal tubular cells (**Figures 4L, M**). This corroborates the severe damage observed in the renal proximal tubules in the previous HE staining (**Figure 2P**).

### 3.4 Spatial transcriptomics validates the pathological significance of lipid metabolism dysregulation

**Figure 5:**
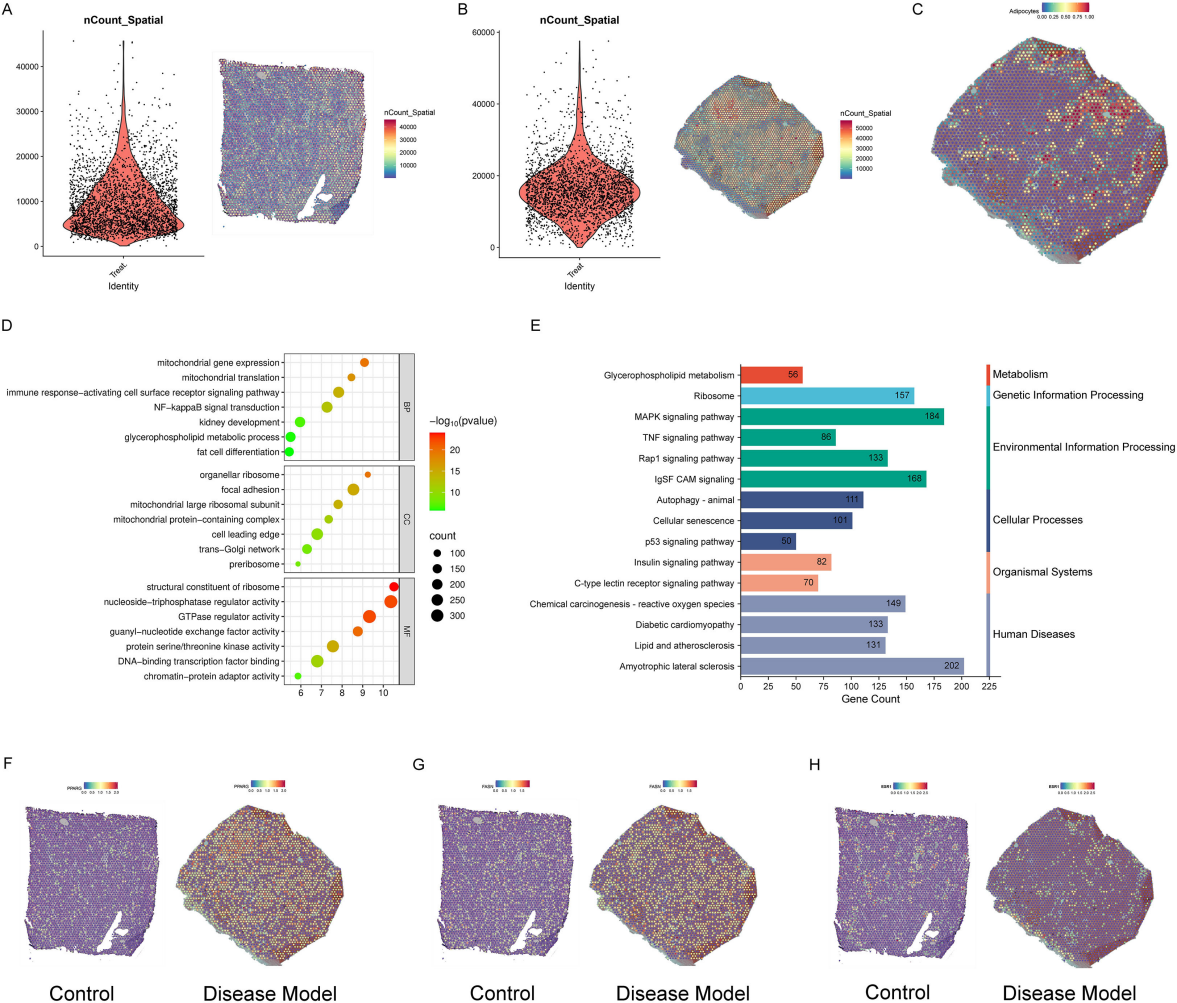
Spatial transcriptomic analysis of renal tissue in a CKM model based on GEO datasets. **(A)** Quality assessment and spatial distribution of transcriptomic data in the healthy control group. **(B)** Quality assessment and spatial distribution of transcriptomic data in the disease group. **(C)** Spatial mapping of adipocyte distribution within the disease group. **(D)** Gene Ontology (GO) enrichment analysis of spatially resolved differentially expressed genes (DEGs). **(E)** Kyoto Encyclopedia of Genes and Genomes (KEGG) pathway enrichment analysis of spatially resolved DEGs. **(F–H)** Spatial expression profiles of PPARG **(F)**, FASN **(G)**, and ESR1 **(H)** across the respective groups.

To validate these findings in spatial context, we performed spatial transcriptomic analyses of renal tissues. Compared with controls, disease samples exhibited marked spatial reorganization of transcriptomic signatures (**Figures 5A, B**, **Supplementary Figure S3**). Tissue deconvolution further revealed abnormal adipocyte-like infiltration and ectopic lipid-associated regions within diseased kidneys (**Figure 5C**). Differential gene analysis demonstrated enrichment of biological processes related to adipocyte proliferation, lipid storage, extracellular remodeling, and inflammatory signaling (**Figure 5D**). KEGG analysis identified significant enrichment in glycerophospholipid metabolism, fatty acid pathways, and lipid and atherosclerosis signaling (**Figure 5E**).

Spatial mapping of hub genes showed robust upregulation of PPARγ and FASN in pathological regions, whereas ESR1 expression was reduced (**Figures 5F–H**). Notably, these signals partially co-localized with adipocyte-enriched niches.

### 3.5 Multi-dataset AI screening identifies actionable metabolic vulnerabilities

**Figure 6:**
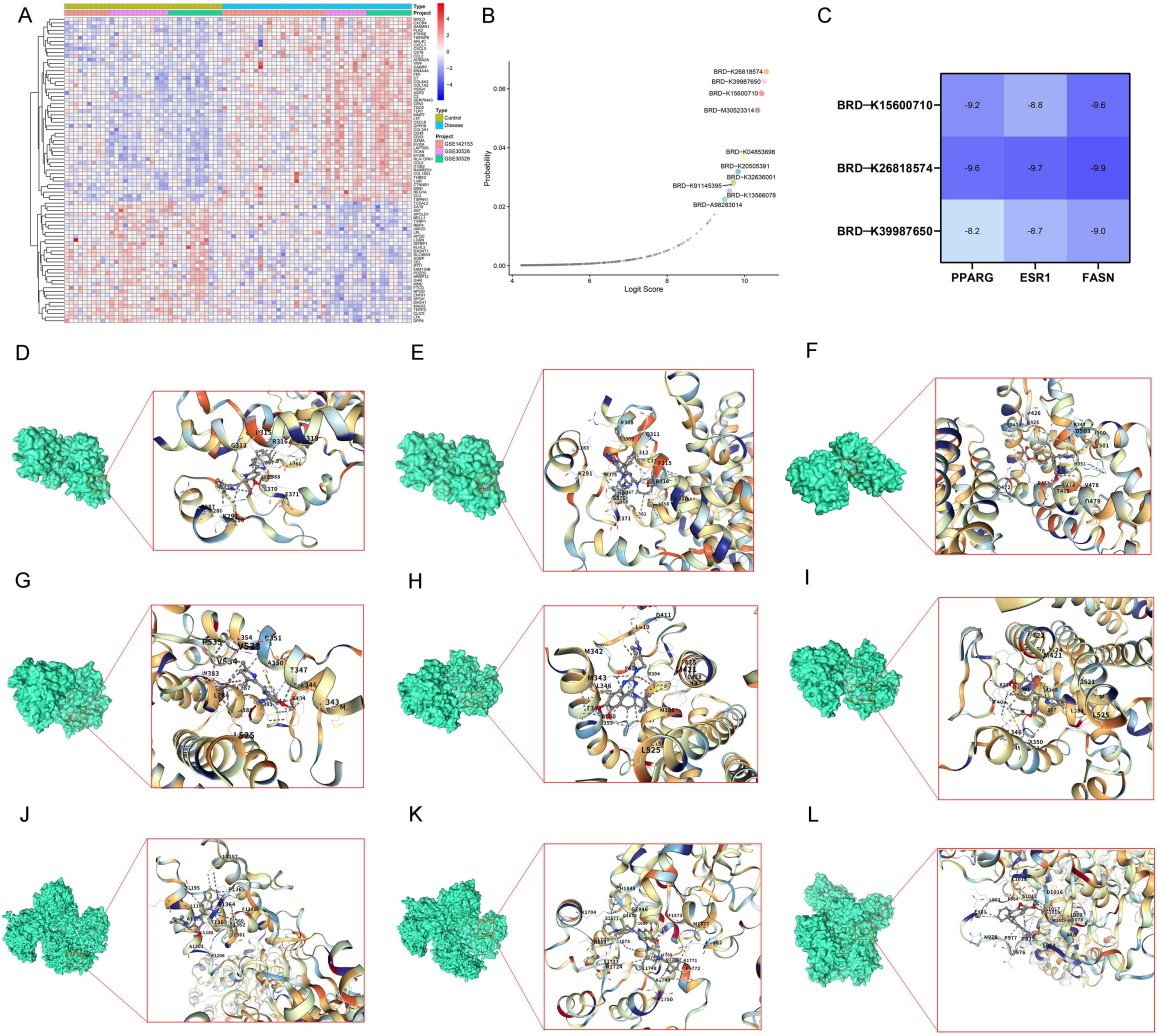
Virtual screening for potential therapeutic compounds based on integrated multi-microarray expression profiling. **(A)** Heatmap illustrating differentially expressed proteins across integrated microarray datasets. **(B)** Visualization of virtual drug screening results. **(C)** Heatmap depicting the binding energy scores from molecular docking of three candidate drugs with core target proteins. **(D–L)** Molecular docking visualizations of BRD-K15600710 **(D–H)**, BRD-K26818574 **(G–I)**, and BRD-K39987650 **(J–L)** with the core targets PPARG, ESR1, and FASN, respectively.

To explore potential therapeutic strategies for CKM, multiple gene expression microarray datasets were integrated to construct a disease-associated differentially expressed gene set (**Figure 6A**). Candidate therapeutic compounds were subsequently screened using the DrugReflector (**Figure 6B**). To further investigate their mechanisms of action, molecular docking analyses were performed between the top three candidate compounds and the core targets PPARγ, ESR1, and FASN. The results showed that: BRD-K15600710 exhibited binding energies of −9.2, −8.8, and −9.6 kcal/mol, respectively (**Figures 6D–F**);BRD-K26818574 showed binding energies of −9.6, −9.7, and −9.9 kcal/mol (**Figures 6G–I**);BRD-K39987650 demonstrated binding energies of −8.2, −8.7, and −9.0 kcal/mol (**Figures 6J–L**). Overall, all candidate compounds displayed strong binding affinities to the core targets. Among them, BRD-K26818574 exhibited the lowest binding energies across all three proteins, indicating its superior potential as a therapeutic candidate for CKM.

## 4 Discussion

This study established a progressive preclinical model that recapitulates the cardiovascular–kidney–metabolic (CKM) syndrome continuum, spanning early metabolic dysfunction to overt cardiorenal injury. By integrating longitudinal phenotyping with multi-omics analyses, we identified maladaptive lipid metabolic remodeling as a prominent molecular feature accompanying CKM progression. Given the profound structural and functional vulnerability of the kidney during CKM progression, our findings further highlight proximal tubular lipid metabolic remodeling as a pathological process associated with systemic metabolic disturbances and progressive cardiorenal injury. Collectively, these findings provide new insights into the progression of CKM.

Although CKM syndrome has recently emerged as a clinically important framework (15), experimental systems that reproduce its multisystem and progressive nature remain limited. Most available models focus on isolated obesity, diabetes, heart failure, or chronic kidney disease phenotypes. In current research on methodological approaches to modeling systemic CKM disorders, the only available strategy involves physical resection of a unilateral kidney (25, 26). Although this approach effectively recapitulates the end-stage pathological state of CKM by inducing excessively severe organic damage within a short timeframe, it poorly reflects the continuous changes that occur during the natural disease course. In contrast, the model established in this study captures the progressive evolution of CKM through sustained induction of nutritional overload and renal pressure, thereby more closely mimicking the authentic pathogenic process. This provides a robust foundation for investigating the progressive factors underlying CKM disease development. Importantly, longitudinal phenotyping revealed a temporal pattern characterized by early metabolic abnormalities followed by progressive cardiorenal deterioration. More specifically, fat accumulation and pathological remodeling in the kidneys became evident at the mid-stage of the disease, whereas significant structural and functional damage to the heart occurred predominantly in the late stage. This temporal pattern is consistent with an early metabolic-dominant stage followed by a later cardiorenal decompensation stage (**Supplementary Figure 1**), providing a useful framework for investigating targeted interventions and therapeutic windows. Furthermore, although hepatic lipid metabolic alterations were evident during earlier metabolic stages, structural injury involving the liver, spleen, and lungs became apparent mainly during advanced disease progression (**Supplementary Figure 4**), suggesting expansion from metabolic dysfunction to broader systemic organ vulnerability. At the same time, the late stage of the illness is accompanied by excessive salivation (**Supplementary Figure 5**) and peripheral lesions that resemble diabetic foot manifestations (**Supplementary Figure 6**). These findings suggest that CKM progression may extend beyond the conventional cardiorenal axis into a broader state of systemic organ vulnerability. This expanded perspective may help explain the substantial heterogeneity of clinical outcomes observed in CKM and underscores the importance of comprehensive systemic phenotyping, together with longitudinal tracking of disease trajectory, for accurately assessing disease progression and therapeutic efficacy.

A key finding of this study is the consistent convergence of multiple orthogonal datasets implicating lipid dysregulation as a shared molecular signature of CKM progression. Network toxicology, single-cell transcriptomics, and spatial transcriptomics independently implicated abnormal lipid handling, fatty acid metabolism, and lipogenic signaling during disease progression. These observations suggest that lipid remodeling is not merely a secondary consequence of obesity or renal dysfunction but may contribute to the transition from systemic metabolic stress to progressive organ injury. This interpretation is aligned with growing evidence that ectopic lipid accumulation can induce mitochondrial dysfunction (27, 28), endoplasmic reticulum stress (29, 30), and inflammasome activation across metabolically active tissues (31, 32). Thus, the biological significance of lipid dysregulation in CKM may extend beyond substrate excess itself, encompassing a broader collapse of cellular stress adaptation and energy homeostasis.

The hub genes identified in our analyses—PPARγ, ESR1, and FASN—are biologically plausible regulators of this process. PPARγ governs adipogenic differentiation, lipid storage, and insulin sensitivity; FASN catalyzes *de novo* lipogenesis; and ESR1 exerts protective metabolic and anti-inflammatory effects in multiple tissues. Coordinated upregulation of PPARγ and FASN together with suppression of ESR1 suggests a maladaptive shift favoring lipid accumulation and ectopic metabolic stress. This triad may therefore represent a biologically relevant regulatory module associated with CKM syndrome.

At the cellular level, our data suggest that renal proximal tubular cells may represent important early responders during CKM progression. These cells have exceptionally high energetic demand and rely heavily on fatty acid oxidation to sustain transport activity. As such, they are particularly vulnerable to mitochondrial dysfunction, impaired lipid utilization, and intracellular lipid accumulation. Our pseudotime and communication analyses suggest that proximal tubular cells may participate in downstream inflammatory and fibrotic remodeling within the renal microenvironment rather than merely serving as passive targets of systemic stress. This concept is increasingly supported by recent studies showing that stressed tubular epithelial cells can adopt secretory phenotypes that recruit immune cells, activate fibroblasts, and propagate chronic injury signals (33). Given the central role of kidney dysfunction in CKM progression, proximal tubular lipotoxicity may represent an early pathological event associated with systemic disease progression.

Notably, the kidney may occupy a more strategic role in CKM biology than traditionally appreciated. Whereas cardiovascular disease is often viewed as the terminal manifestation and obesity as the initiating insult, the kidney functions as both a metabolic sensor and endocrine regulator. Tubular injury can alter sodium handling, erythropoietin balance (34), uremic toxin accumulation, RAAS activation (35), and gluconeogenic flux (36), thereby influencing blood pressure (37), insulin sensitivity, inflammation, and myocardial workload (38). Under this framework, renal metabolic dysfunction may contribute to the transition from compensated metabolic disease to overt multisystem failure. This possibility may help explain why therapies that preserve kidney function often produce disproportionately strong cardiovascular benefits.

Spatial transcriptomics further strengthened this interpretation by revealing localized pathological niches characterized by adipocyte-like infiltration, lipogenic gene activation, and tissue remodeling. These findings are consistent with emerging evidence that ectopic lipid deposition promotes fibrosis, oxidative stress, and capillary rarefaction in chronic kidney disease (39, 40). The spatial co-localization of hub genes with abnormal lipid-associated regions highlights the importance of microenvironmental context in CKM pathology and suggests that regional metabolic heterogeneity may influence disease severity.

Finally, integrative virtual drug screening identified BRD-K26818574 as a candidate compound targeting the core lipid regulatory network. The binding energies of this compound to its cognate target are remarkably low (all below –9 kcal/mol), implying a high likelihood of direct binding and subsequent functional modulation. Its predicted multi-target interactions with PPARγ, ESR1, and FASN suggest a network-based mechanism of action, which may be particularly relevant given the systems-level nature of CKM pathophysiology, where pathway redundancy and compensatory signaling limit single-target interventions.

This study has several limitations. First, although the proposed model recapitulates key pathological features and the progressive nature of CKM syndrome, it cannot fully represent the heterogeneity and clinical complexity of human disease. Second, the single-cell and spatial transcriptomic analyses were performed using publicly available external datasets rather than samples derived from the current experimental cohort. Although these external datasets provided independent validation support for the molecular signatures identified in our study, the lack of transcriptomic analyses directly generated from our own experimental samples represents a limitation. Future studies incorporating single-cell and spatial transcriptomic profiling of samples from the established model are warranted to further validate and refine the identified molecular mechanisms. Third, this study focused on convergent molecular alterations identified through multi-omics integration and did not comprehensively investigate other established CKM-related pathways, including RAAS activation, inflammation, oxidative stress, sympathetic activation, and mitochondrial dysfunction. Future studies integrating these pathways with multi-omics approaches are needed to construct a more comprehensive regulatory framework. Finally, candidate therapeutic compounds identified by in silico screening require further validation *in vitro* and *in vivo* to confirm their efficacy and mechanisms of action.

In conclusion, we establish a translational framework for modeling CKM syndrome and demonstrate that renal lipid metabolic reprogramming is closely linked to systemic disease progression. By integrating animal phenotyping with systems biology and spatially resolved transcriptomics, this study identifies lipid metabolic maladaptation as a potentially actionable pathological process associated with CKM syndrome and provides a framework for exploring precision therapeutic strategies.

## 5 Conclusion

A progressive rat model successfully reproduced staged CKM syndrome progression. Multi-omics analyses identified renal lipotoxic remodeling centered on the PPARγ–ESR1–FASN network as a key pathological feature. These findings provide mechanistic insight into CKM pathogenesis and support the development of metabolism-targeted therapeutic strategies for future translational applications.

## Data Availability

The original contributions presented in the study are included in the article/**Supplementary Material**. Further inquiries can be directed to the corresponding author.
