# Supplementary material for: A novel continuous-progression CKM model combined with multi-omics data integration identifies lipid metabolic drivers of disease progression
Source: Front Endocrinol (Lausanne). 2026 Jul 29;17:1910505. doi: 10.3389/fendo.2026.1910505 (PMC13461501; doi:10.3389/fendo.2026.1910505)
Supplement: Supplementary file 1 [file SupplementaryFile1.docx]

**A Novel Continuous-Progression CKM Model Combined with Multi-Omics Data Integration Identifies Lipid Metabolic Drivers of Disease Progression**

**Supplementary Figure**

**Supplementary Fig. S1 Progressive changes in the heart and kidney.**

(A) PCA analysis of kidney tissues at indicated time points.(B) Changes in cardiac fractional shortening (FS) at selected time points.(C) Changes in cardiac ejection fraction (EF) at selected time points.(D) Changes in renal triglyceride (TG) content at selected time points.(E) H&E staining of heart and kidney sections at selected time points.

**Supplementary Fig. S2 Quality control, dimensionality reduction clustering, and cell-cell communication analysis of single-cell transcriptomic data**

A. Scatter plot for quality control of single-cell data. B. Distribution of nFeature_RNA, nCount_RNA, and mitochondrial gene proportion across samples. C. Heatmap of genes associated with the first four principal components from PCA. D. JackStraw analysis plot showing the significance of principal components. E. t-SNE dimensionality reduction and clustering plot based on cell clusters. F. Proportional composition of ligand-receptor interaction sources and types derived from cell-cell communication databases. G. Cell-cell communication network diagram based on ligand-receptor interaction strength. H. Bubble plot of significant ligand-receptor pairs between different cell types.

**Supplementary Fig. S3 Spatial mapping of each cell type**

The model group shows obvious aggregation of specific cell types, and the cellular microenvironment has changed.

**Supplementary Fig. S4 H&E staining of Liver , Lung and Spleen sections at selected time points.**

**Supplementary Fig. S5 Photos of drooling symptoms at the end point of the model**

The rats show obvious continuous drooling, and small bleeding spots appear around the mouth.

**Supplementary Fig. S6 Photos of suspected diabetic foot symptoms at model endpoint**

Obvious hemorrhagic plaques appeared on the rat's foot and were not easy to heal, suspected to be symptoms related to diabetic foot
